# Machine Learning-Driven QSRR Modeling of Albumin Binding in Fluoroquinolones: An SVR Approach Supported by HSA Chromatography

**DOI:** 10.3390/ijms27083700

**Published:** 2026-04-21

**Authors:** Yash Raj Singh, Wiktor Nisterenko, Joanna Fedorowicz, Jarosław Sączewski, Daniel Szulczyk, Katarzyna Ewa Greber, Wiesław Sawicki, Krzesimir Ciura

**Affiliations:** 1 Department of Physical Chemistry, Medical University of Gdansk, Al. Gen. J. Hallera 107, 80-416 Gdansk, Poland; yashr.singh@gumed.edu.pl (Y.R.S.); wiktor.nisterenko@gumed.edu.pl (W.N.); katarzyna.greber@gumed.edu.pl (K.E.G.); wieslaw.sawicki@gumed.edu.pl (W.S.); 2 Department of Chemical Technology of Drugs, Faculty of Pharmacy, Medical University of Gdansk, Al. Gen. J. Hallera 107, 80-416 Gdansk, Poland; joanna.fedorowicz@gumed.edu.pl; 3 Department of Organic Chemistry, Faculty of Pharmacy, Medical University of Gdansk, Al. Gen. J. Hallera 107, 80-416 Gdansk, Poland; jaroslaw.saczewski@gumed.edu.pl; 4 Chair and Department of Biochemistry, Medical University of Warsaw, 02-097 Warsaw, Poland; daniel.szulczyk@wum.edu.pl; 5 Laboratory of Environmental Chemoinformatics, Faculty of Chemistry, University of Gdansk, 63 Wita Stwosza Street, 80-308 Gdansk, Poland

**Keywords:** human serum albumin, fluoroquinolones, QSRR, support vector regression, biomimetic chromatography, plasma protein binding

## Abstract

Human serum albumin (HSA) binding critically influences drug distribution and pharmacokinetics. In this study, HSA affinity chromatography was integrated with machine-learning-based quantitative structure–retention relationship (QSRR) modeling to elucidate structural determinants of albumin binding in a library of 115 fluoroquinolone (FQs) derivatives. Experimentally determined log*k*_HSA_ values were obtained using biomimetic chromatography, and these were then used as modelling endpoints. Following descriptor reduction via Least Absolute Shrinkage and Selection Operator (LASSO) and systematic benchmarking of 42 regression algorithms, support vector regression (SVR) and nu-support vector regression (ν-SVR) with radial basis function kernels demonstrated superior predictive performance. A parsimonious 12-descriptor ν-SVR model achieved strong calibration and validation metrics (R^2^ = 0.916, Q^2^_test_ = 0.823, concordance correlation coefficient (CCC) = 0.899) and satisfied Organisation for Economic Co-operation and Development (OECD) criteria, including applicability domain assessment. Shapley Additive exPlanations (SHAP)-based interpretation revealed that albumin binding is governed by a balance between hydrophobic surface area and distributed electronic properties, whereas excessive localized polarity and quaternary ammonium functionalities reduce affinity. This experimentally anchored and interpretable modeling framework provides mechanistic insight into HSA binding in fluoroquinolones and offers a robust tool for rational pharmacokinetic optimization. Furthermore, in order to make the model easily accessible to users, we have packaged it in the form of an online application.

## 1. Introduction

Fluoroquinolones (FQs) represent a well-established class of antibacterial agents characterized by broad-spectrum activity and a generally favorable safety profile [1,2]. In addition to their antimicrobial efficacy, numerous studies have demonstrated that FQs and structurally related scaffolds exhibit diverse biological activities, including anticancer, antiviral, and anti-inflammatory effects [3,4,5]. This pharmacological versatility has rendered the quinolone core a privileged scaffold in medicinal chemistry and a valuable starting point for the development of novel therapeutic agents [6,7].

Despite their clinical utility, the pharmacokinetic performance of many FQs remains suboptimal. Their zwitterionic nature at physiological pH, limited aqueous solubility, and susceptibility to active efflux transporters such as P-glycoprotein can significantly restrict membrane permeability, intracellular accumulation, and tissue distribution [8,9]. Because the therapeutic efficacy of anti-infective agents critically depends on achieving adequate concentrations at the site of infection, rational optimization of ADME-related properties remains a central challenge in the development of new FQ derivatives.

Among the key determinants of drug disposition, plasma protein binding plays a pivotal role [10]. Human serum albumin (HSA), the most abundant plasma protein, functions as a major transport protein for endogenous compounds and xenobiotics [11,12,13], including FQs, for which albumin has been identified as the predominant binding protein [14]. Binding to HSA influences drug distribution, free (pharmacologically active) fraction, half-life, and potential drug–drug interactions. Importantly, the extent of albumin binding has been shown to directly affect antimicrobial efficacy, as elevated albumin levels significantly reduce the bactericidal activity of fluoroquinolones such as moxifloxacin and trovafloxacin [15]. Even moderate structural modifications can substantially alter albumin affinity through changes in hydrophobic surface area, hydrogen-bonding capacity, conformational flexibility, or overall molecular polarity. Indeed, computational studies have demonstrated that modifications at specific positions of the quinolone core can markedly influence protein binding rates [16]. However, despite extensive synthetic efforts aimed at diversifying the FQ scaffold, the quantitative relationship between structural variation and HSA binding has not been systematically elucidated for chemically diverse FQ libraries. In particular, an integrated chemometric framework linking molecular descriptors with experimentally determined albumin affinity remains underdeveloped.

Chromatographic techniques provide efficient and reproducible tools for probing drug–biomolecule interactions. Reversed-phase liquid chromatography is widely used for lipophilicity assessment, whereas biomimetic stationary phases extend chromatographic analysis toward physiologically relevant chemical interactions [17,18,19]. In particular, HSA affinity chromatography offers a rapid and high-throughput surrogate for estimating albumin binding under controlled experimental conditions [20]. When combined with computational modeling approaches, chromatographic retention parameters can serve as experimentally anchored endpoints for quantitative structure–retention relationship (QSRR) analysis [21,22]. Recent advances in machine learning (ML) have substantially expanded the scope and predictive power of QSAR/QSRR modeling beyond classical linear approaches such as multiple linear regression or partial least squares [23,24]. Methods based on neighborhood principles, decision tree ensembles, and kernel-based techniques have demonstrated the ability to capture nonlinear structure–property relationships, frequently outperforming linear models in predictive accuracy and generalization [25,26]. However, the systematic application of ML-driven QSRR to model albumin binding within structurally diverse fluoroquinolone libraries has not been reported to date. In the present study, we integrated HSA affinity chromatography with advanced QSRR modeling to investigate how structural modifications within a chemically diverse library of fluoroquinolone derivatives influence albumin binding. The dataset comprised 115 compounds, including marketed FQ drugs and newly synthesized derivatives containing the quinolone core. Multiple regression algorithms were systematically compared to identify the optimal modeling strategy. Following model selection, hyperparameter optimization and rigorous internal and external validation were performed. The final models were assessed according to the OECD principles for QSAR validation, including a defined applicability domain, goodness-of-fit, robustness, and predictive performance.

This integrative experimental–computational approach aims to provide mechanistic insight into the structural determinants of HSA binding within the FQ class and to support the rational design of derivatives with improved pharmacokinetic profiles. Furthermore, in order to make the model easily accessible to users, we have packaged it in the form of an online application https://fqs-qsar.streamlit.app/.

## 2. Results and Discussion

**Figure 1:**
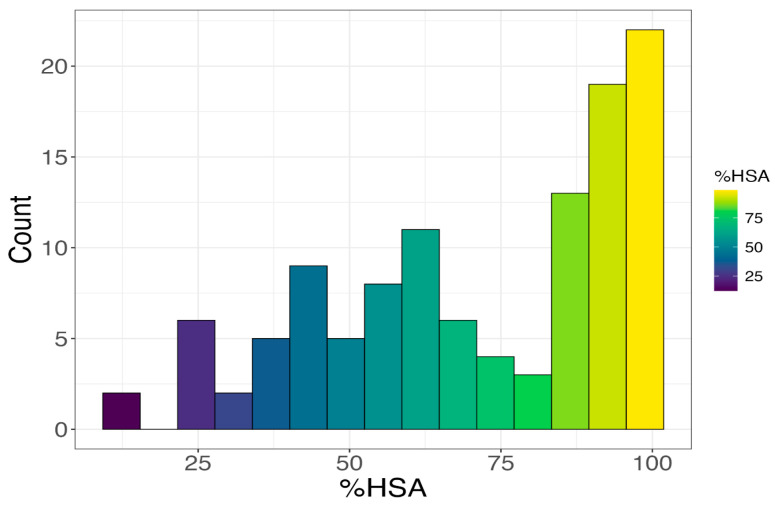
Distribution of human serum albumin binding (HSA%) across the investigated fluoroquinolone derivatives.

The distribution of experimentally determined HSA binding across the investigated fluoroquinolone library is presented in Figure 1. The histogram reveals a broad and continuous range of albumin affinity, spanning approximately from 12.8% to nearly 99.2%. Although a substantial fraction of compounds exhibits high binding (>80%), a considerable number populate the intermediate range (40–70%), and several derivatives display markedly reduced affinity. This wide dispersion confirms that the dataset encompasses compounds with substantially different albumin-binding profiles, providing an appropriate dynamic range for quantitative structure–retention modeling.

**Figure 2:**
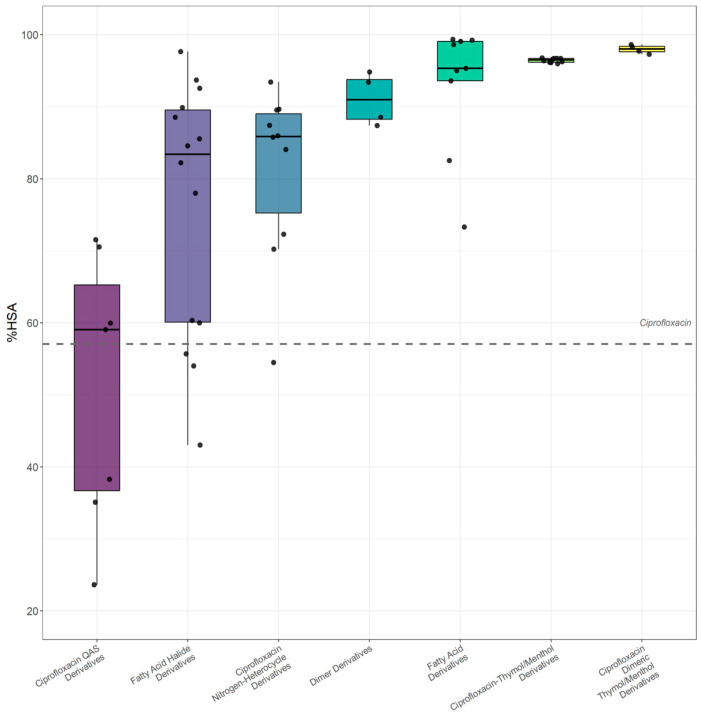
Distribution of HSA binding (HSA%) across structural subclasses of ciprofloxacin derivatives presented as box plots. The dashed line represents the albumin binding level of the parent ciprofloxacin.

The broad variability of HSA binding observed across the entire dataset becomes particularly evident within the largest and structurally coherent subgroup, namely the ciprofloxacin derivatives (n = 58). These compounds were categorized according to the type of structural modification introduced into the ciprofloxacin core, including fatty acid and fatty acid halide derivatives, heterocyclic conjugates, thymol and menthol conjugates, quaternary ammonium salt analogues, as well as dimeric derivatives and their thymol and menthol conjugates. As illustrated in Figure 2, albumin binding strongly depends on the nature of the modification. Conjugates with thymol or menthol, together with their dimeric analogues, exhibit consistently high HSA% values, whereas fatty acid and fatty acid halide derivatives show markedly greater dispersion, with affinities ranging from values comparable to the parent drug to substantially lower levels. Quaternary ammonium salt analogues generally display binding similar to or slightly below that of ciprofloxacin. Notably, heterocyclic derivatives span a particularly wide range of HSA% values, indicating that relatively subtle changes in heterocycle type and linker architecture substantially influence albumin affinity. Overall, these findings demonstrate that even within a common quinolone scaffold, targeted structural modifications translate into pronounced differences in HSA binding, thereby providing a clear rationale for subsequent quantitative modeling.

To quantitatively describe the observed variability in HSA binding, QSRR modeling was performed using experimentally determined log*k*_HSA_ values as the response variable. Although HSA% values are presented for descriptive and visualization purposes (Figure 2), model development was based on log*k*_HSA_ to avoid regression on bounded percentage data and to prevent distortion introduced by the nonlinear transformation of log*k*_HSA_ into percentage values.

The dataset was partitioned into training and external test sets using the Kennard–Stone algorithm, ensuring representative coverage of the chemical descriptor space in both subsets. This stratified distribution of compounds across the training and test sets provided a suitable basis for reliable model development and unbiased external validation.

**Figure 3:**
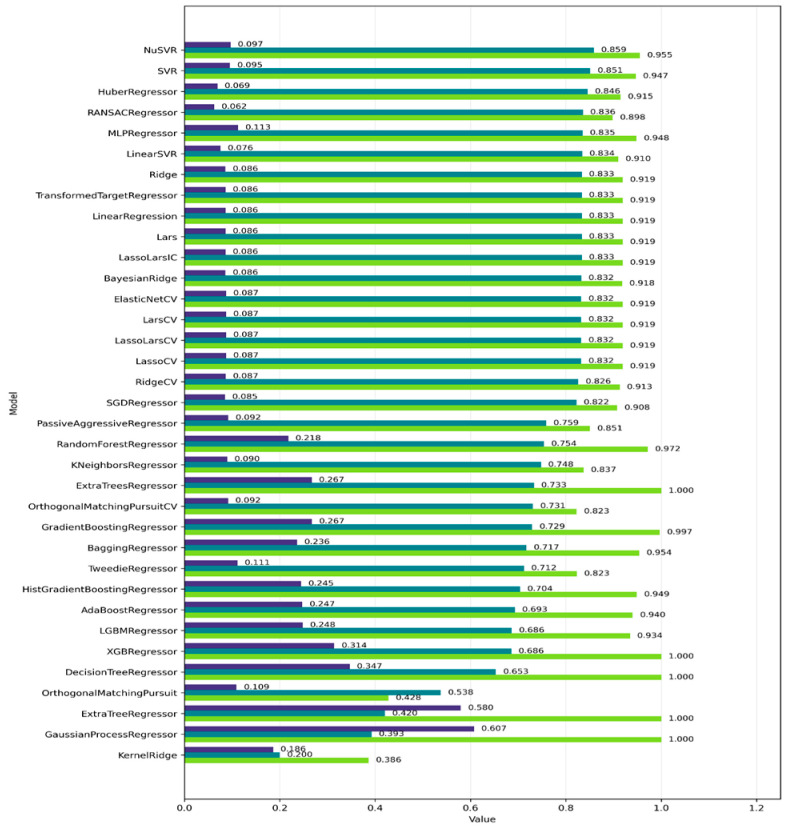
Comparative performance of the evaluated regression models for HSA binding prediction. For each model, the bars represent the training R^2^ (light green), test R^2^ (dark green), and the train-test performance gap (ΔR^2^ = R^2^_train_ − R^2^_test_; purple), used as an indicator of overfitting.

The initial phase of QSRR modeling focused on identifying an appropriate regression algorithm. A total of 42 machine-learning regressors were evaluated under identical preprocessing conditions and default hyperparameter settings to ensure consistent benchmarking. Prior descriptor selection was performed using LASSO regression, reducing the descriptor space to a maximum of 17 variables (Table S5) in accordance with the Topliss–Costello rule. Based on this reduced descriptor pool, the evaluated algorithms were ranked according to their training and test R^2^ values, RMSE, and the train–test performance gap. The complete set of metrics for all models is summarized in Figure 3.

This screening provided a comprehensive overview of predictive accuracy and generalization behavior across different regression paradigms. Linear models exhibited highly consistent performance, with comparable training and test R^2^ values across ridge, lasso, elastic net, and related approaches. This observation is likely influenced by the prior descriptor selection step based on linear LASSO regression, which favors descriptors with predominantly linear contributions to the response variable.

In contrast, tree-based and ensemble methods demonstrated a clear tendency toward overfitting. Several of these models achieved near-perfect training R^2^ values while showing substantially lower performance on the independent test set, indicating limited extrapolation capability beyond the training domain.

Among all evaluated algorithms, support vector regression (SVR) and ν-SVR employing a radial basis function (RBF) kernel achieved the most favorable balance between training accuracy and test performance. This finding is consistent with the established effectiveness of SVR in QSAR and QSRR modeling, where its ability to handle nonlinear structure–property relationships through kernel-based transformations has been widely recognized [27]. In the chromatographic context, SVR has demonstrated strong predictive performance for retention modeling of structurally diverse analytes, including quinolone antibiotics [28,29]. Both approaches demonstrated strong predictive power while maintaining a relatively small train–test performance gap, indicative of stable generalization. The consistent top-ranking performance of both SVR variants suggests that the relationship between molecular descriptors and HSA binding is not strictly linear and contains systematic nonlinear components effectively captured by kernel-based methods.

Given their superior and consistent performance, SVR and ν-SVR were selected for further refinement and optimization. Hyperparameter tuning was conducted within the predefined cross-validation framework to identify configurations maximizing predictive stability. Following initial optimization based on the 18-descriptor subset, feature contributions were examined using SHAP values. Descriptors showing consistently negligible impact on model predictions, often affecting only isolated observations, were removed in order to obtain a more parsimonious and stable model.

The reduced descriptor set comprising 12 variables (Table S6) was subsequently used for re-optimization and full validation of both SVR variants according to the same evaluation protocol. The predictive performance of the optimized SVR and ν-SVR models is summarized in Table 1. Both models demonstrated strong fitting ability, with R^2^ values of 0.901 (SVR) and 0.916 (ν-SVR), indicating that over 90% of the variance in HSA binding was explained within the training set. The corresponding RMSE values (0.206 and 0.190, respectively) confirm low residual error and high internal consistency.

Cross-validation results further support model robustness. The Q^2^_CV_ values of 0.818 for SVR and 0.811 for ν-SVR indicate stable predictive behavior under internal resampling, with only a moderate decrease relative to calibration performance. The similarity between R^2^ and Q^2^_CV_ suggests limited overfitting and good structural stability of the models.

External validation on the independent test set confirms strong generalization capability. The ν-SVR model achieved slightly superior predictive performance (Q^2^_test_ = 0.823, RMSE_test_ = 0.238) compared to SVR (Q^2^_test_ = 0.800, RMSE_test_ = 0.252). This trend is consistently reflected across Q^2^_F1–F3_ statistics and CCC, where ν-SVR reached CCC = 0.899 versus 0.882 for SVR.

Overall, both models meet commonly accepted criteria for externally predictive QSRR models, with ν-SVR showing marginally improved predictive accuracy and concordance. The slightly better performance of ν-SVR may be attributed to its alternative regularization formulation, where the ν parameter explicitly controls the fraction of support vectors and training errors, potentially leading to a more flexible bias–variance balance compared to standard ε-SVR. Given its slightly superior generalization metrics, the ν-SVR configuration was selected as the final model for further analysis.

**Figure 4:**
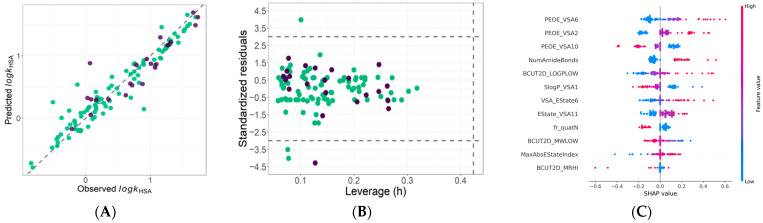
Observed versus predicted HSA% values (**A**), Williams plot for applicability domain assessment (**B**), and SHAP summary plot illustrating descriptor contributions (**C**) for the final optimized 12-descriptor ν-SVR model. In panels (**A**,**B**), green dots represent the training set and purple dots represent the test set. In panel (**B**), the grey dashed horizontal lines indicate the standardized residual limits at +3 and −3, while the grey dashed vertical line indicates the critical leverage threshold (h*).

The robustness of the final model is further supported by the observed versus predicted plot (Figure 4A), which demonstrates close agreement between experimental and predicted values for both training and external test compounds, without systematic deviation or trend bias.

The applicability domain (AD), assessed using the leverage approach and visualized in the Williams plot (Figure 4B), indicates that the vast majority of compounds fall within the defined reliability limits (h < h* and standardized residuals within ±3). Only four molecules lie outside the structural applicability domain (three from the training set and one from the test set), corresponding to 3.4% of the entire dataset. No compounds exceeded the standardized residual threshold. These results confirm the structural stability and predictive reliability of the final ν-SVR model.

In addition to statistical robustness and external predictivity, compliance with the OECD principles for QSAR/QSRR validation requires the provision of a mechanistic interpretation, whenever possible. To address this criterion, model interpretability was assessed using Shapley Additive exPlanations (SHAP), a game-theory-based framework that quantifies the contribution of each descriptor to individual predictions.

SHAP values decompose the model output into additive feature contributions relative to a baseline expectation, thereby enabling both global and local interpretation of descriptor influence. This approach allows identification of the most influential molecular properties governing HSA binding and clarifies the direction (positive or negative) of their impact on model predictions.

The SHAP-based interpretation (Figure 4C) indicates that HSA binding in the investigated FQs is governed by a complex interplay between hydrophobic surface area and spatially distributed electronic properties. Descriptors belonging to the PEOE_VSA family, particularly PEOE_VSA2 and PEOE_VSA6, exhibit predominantly positive SHAP contributions at higher values, suggesting that moderately polarized van der Waals surface regions promote albumin affinity. This trend is consistent with the role of distributed partial charges facilitating favorable interactions within the HSA binding pocket.

In contrast, increased contributions from highly polarized surface fragments (e.g., PEOE_VSA10) and the presence of quaternary ammonium functionalities (fr_quatN) are associated with reduced predicted binding, indicating that excessive localized polarity or strong cationic character may disfavor interaction with albumin. Importantly, quaternary ammonium salt derivatives constitute a substantial fraction of the investigated dataset (21%), and their consistent negative SHAP contributions underline the robustness of this signal. The observed trend is therefore not driven by isolated outliers but reflects a systematic structural effect within a well-represented subclass.

Lipophilicity-related descriptors, including BCUT2D_LOGPLOW, generally show positive contributions at elevated values, supporting the importance of hydrophobic interactions in stabilizing drug–albumin complexes. However, certain surface-partitioned lipophilicity descriptors (e.g., SlogP_VSA1) display a more nuanced pattern, suggesting that not all hydrophobic surface contributions uniformly enhance binding.

Overall, the SHAP analysis supports a mechanistic model in which optimal HSA binding arises from a balanced combination of hydrophobic surface area, moderate polarity, and favorable electronic distribution, whereas excessive localized charge or disproportionate surface characteristics may reduce albumin affinity.

**Figure 5:**
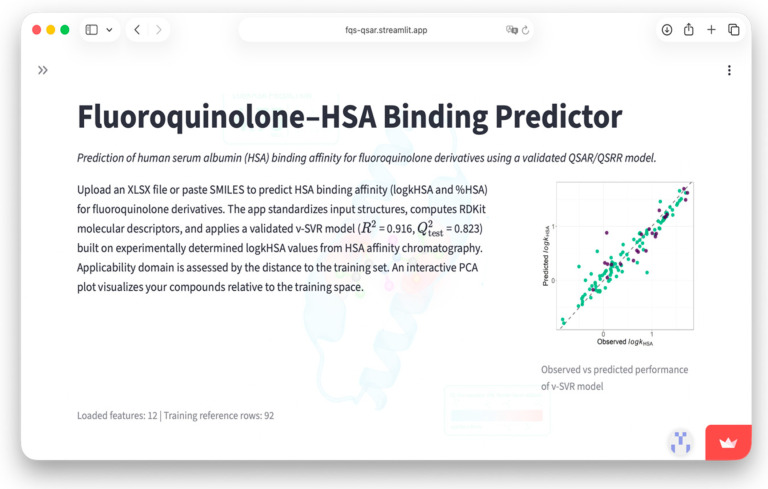
Graphical user interface of the FQs QSAR web application for prediction of HSA binding.

Furthermore, to enhance the translational value of the developed QSRR framework and facilitate its practical use, the final validated ν-SVR model has been deployed as an openly accessible web application (https://fqs-qsar.streamlit.app/). The platform enables rapid in silico prediction of HSA binding for FQs derivatives based on user-provided SMILES strings or batch XLSX input. Submitted structures are automatically standardized, the required RDKit descriptors are computed, and HSA ng is predicted using the optimized 12-descriptor model. The application additionally provides the distance to the training space, allowing users to assess whether predictions fall within the defined applicability domain. A representative view of the graphical user interface implementing the predictive workflow is presented in Figure 5.

## 3. Materials and Methods

### 3.1. Chemical Reagents

Dimethyl sulfoxide (DMSO) and 2-propanol (HPLC grade) were purchased from Sigma-Aldrich (Steinheim, Germany). Ammonium acetate, used for the preparation of the mobile phase buffer, was obtained from VWR International (Leuven, Belgium). Ultrapure water used for mobile phase preparation was produced with a Millipore Direct-Q 3 UV purification system (Millipore Corporation, Bedford, MA, USA). All solvents and reagents were of HPLC grade and were used without further purification. Reference compounds employed as calibration standards for HSA binding determination are listed in Table S1, together with detailed information on their suppliers.

### 3.2. Analytes

The sources of the commercially available fluoroquinolones (FQs) are listed in Table S2. The synthesis, purification, and structural characterization of the remaining FQ derivatives have been described previously. SMILES codes and two-dimensional (2D) chemical structures of all investigated compounds are provided in Table S2 (Supplementary Information). Prior to chromatographic analysis, all compounds were dissolved in dimethyl sulfoxide (DMSO) to obtain stock solutions at a concentration of 1 mg/mL. The stock solutions were stored at 2–8 °C and diluted with the appropriate mobile phase to a final concentration of 100 µg/mL immediately before HPLC analysis. Between analyses, stock solutions were kept at 2–8 °C.

### 3.3. Chromatographic Analysis

All chromatographic experiments were performed using a Prominence LC-2030C 3D HPLC system (Shimadzu, Kyoto, Japan) controlled by LabSolutions software (version 5.90, Shimadzu, Japan) and equipped with a diode-array detector (DAD). Albumin affinity was evaluated using a CHIRALPAK^®^ HSA column (5 μm, 4.0 × 50 mm) fitted with a compatible guard column (Daicel Chiral Technologies, West Chester, PA, USA). The mobile phase consisted of 50 mM ammonium acetate buffer (pH 7.4, solvent A) and 2-propanol (solvent B). The flow rate was set at 0.9 mL/min, and a linear gradient elution program was applied as follows: 0–5 min, 0–30% B; 5–10 min, held at 30% B; 10–11 min, returned to 0% B; followed by re-equilibration to give a total run time of 14 min. The column temperature was maintained at 30 °C to ensure retention time reproducibility while minimizing thermal degradation of the immobilized HSA stationary phase. A linear calibration model constructed using the reference compounds listed in Table S1 was employed to calculate the chromatographic retention parameter (log*k*_HSA_). The obtained log*k*_HSA_ values were subsequently transformed into the percentage of human serum albumin binding (HSA%) according to Equation (1):

(1) $$HSA\%\;=\frac{101\cdot 10^{{logk}_{HSA}}}{1+10^{{logk}_{HSA}}}$$

All measurements were performed in duplicate, and mean retention times were used for further analysis. The injection volume was 10 µL. The retention data for all investigated fluoroquinolone derivatives are provided in Table S3 (Supplementary Information).

### 3.4. Molecular Descriptors

Molecular descriptors were calculated from standardized SMILES representations of all compounds using the RDKit library (version 2025.09.3) implemented in Python (version 3.11.9). Prior to descriptor calculation, chemical structures were standardized by removing salts, normalizing functional groups, and generating canonical SMILES. The initial descriptor set consisted of RDKit’s standard two-dimensional (2D) molecular descriptors, including physicochemical, topological, constitutional, and fragment-based parameters. All calculated descriptors were compiled into a single descriptor matrix (Table S4).

### 3.5. QSRR Modeling

#### 3.5.1. Data Set Preparation and Splitting

The dataset was divided into training and test subsets using the Kennard–Stone algorithm implemented in Python (version 3.11.9). Pairwise Euclidean distances were calculated using the pairwise_distances function from the scikit-learn library (version 1.7.2). An 80/20 split was applied, with 80% of the compounds assigned to the training set and 20% reserved as an external test set. The training set was used for descriptor selection, model fitting, hyperparameter optimization, and cross-validation. The test set was used exclusively for external validation.

#### 3.5.2. Descriptor Reduction

Descriptor selection was performed on the training set using the Least Absolute Shrinkage and Selection Operator (LASSO) regression via the scikit-learn library (version 1.8.0) in Python (version 3.11.9). The number of retained descriptors was limited according to the Topliss–Costello criterion [30], maintaining a minimum ratio of five training compounds per descriptor. A final subset of 17 descriptors was selected. The selected descriptor subset was used in all subsequent modeling steps.

#### 3.5.3. Initial Model Screening

Regression algorithms were screened using the LazyPredict library (version v0.2.16) in Python (version 3.11.9). All models were trained on the training set and evaluated using the predefined training/test split. Performance was assessed using the coefficient of determination (R^2^) calculated for both training and test sets. The difference between training and test performance (ΔR^2^ = R^2^_train_ − R^2^_test_) was also calculated. The screening results indicated that SVR and ν SVR were the two best-performing approaches, showing only minor differences in predictive accuracy and generalization gap; therefore, both models were carried forward for further evaluation and model refinement.

#### 3.5.4. Model Optimization and Validation

SVR and ν SVR were applied to predict log*k*_HSA_ using the reduced descriptor set obtained from LASSO selection. Hyperparameter optimization was performed by exhaustive grid search combined with five-fold cross-validation on the training subset. All descriptors were standardized using z-score scaling implemented within the modeling pipeline. Model selection was based on the mean cross-validated coefficient of determination (R^2^). The SVR model was implemented using a radial basis function (RBF) kernel. The hyperparameter search space included C (0.1, 1, 10, 100), ε (0.01, 0.05, 0.1, 0.2), and γ (“scale”, 0.01, 0.05, 0.1, 0.2). For ν SVR, the model was likewise restricted to an RBF kernel, and the hyperparameter grid included ν (0.2, 0.3, 0.4, 0.5, 0.6, 0.7) together with C (0.1, 1, 10, 100) and γ (“scale”, 0.01, 0.05, 0.1, 0.2).

Following optimization, the model was refitted on the full training subset using the selected hyperparameters and evaluated on the independent test subset. Model performance was assessed using the coefficient of determination (R^2^), root mean square error (RMSE), and mean absolute error (MAE) for the training set, cross-validated predictions, and the external test set. Model interpretation was conducted within an out-of-fold cross-validation framework. In each fold, the optimized pipeline was refitted using the fold-specific training portion. Permutation feature importance was calculated by measuring the decrease in R^2^ under repeated feature permutation (50 repetitions). Fold-level importance scores were averaged across folds. Shapley Additive exPlanations (SHAP) values were computed using a kernel-based explainer within the same out-of-fold framework [31]. For each fold, SHAP values were estimated for compounds in the held-out portion, and global feature importance was summarized as the mean absolute SHAP value per descriptor aggregated across folds.

Next, based on the SHAP importance values, descriptors exhibiting negligible contribution to model predictions were removed for both SVR and ν-SVR. This refinement yielded a reduced descriptor subset comprising 12 descriptors. Each model was subsequently refitted using the same hyperparameter optimization and validation protocol as described above. The final reported models correspond to these 12-descriptor configurations.

#### 3.5.5. Model Evaluation and Performance Metrics

Model performance was evaluated using the coefficient of determination (R^2^), root mean square error of calibration (RMSE_C_), cross-validated coefficient (Q^2^_CV_), and root mean square error of cross-validation (RMSE_CV_). External validation was performed using Q^2^_F1_, Q^2^_F2_, Q^2^_F3_, the concordance correlation coefficient (CCC), and the root mean square error of prediction (RMSE_P_).

$$R^{2}=1-\frac{\sum_{i=1}^{n}(y_{i}-{\hat{y}}_{i}{)}^{2}}{\sum_{i=1}^{n}(y_{i}-\bar{y}{)}^{2}}$$

$$MAE=\frac{1}{n}\sum_{i=1}^{n}\left|y_{i}-{\hat{y}}_{i}\right|$$

$$RMSE=\sqrt{\frac{1}{n}\sum_{i=1}^{n}(y_{i}-{\hat{y}}_{i}{)}^{2}}$$

$$Q_{F1}^{2}=1-\frac{\sum_{i=1}^{n_{test}}(y_{i}-{\hat{y}}_{i}{)}^{2}}{\sum_{i=1}^{n_{test}}(y_{i}-y_{train}{)}^{2}}$$

$$Q_{F2}^{2}=1-\frac{\sum_{i=1}^{n_{test}}(y_{i}-{\hat{y}}_{i}{)}^{2}}{\sum_{i=1}^{n_{test}}(y_{i}-{\bar{y}}_{test}{)}^{2}}$$

$$Q_{F3}^{2}=1-\frac{\frac{1}{n_{test}}\sum_{i=1}^{n_{test}}(y_{i}-{\hat{y}}_{i}{)}^{2}}{\frac{1}{n_{train}}\sum_{i=1}^{n_{train}}(y_{i}-{\bar{y}}_{train}{)}^{2}}$$

$$CCC=\frac{2S_{y\hat{y}}}{S_{yy}+S_{\hat{y}\hat{y}}+{n\left(\bar{y}-\hat{\bar{y}}\right)}^{2}},$$

where

$$\bar{y}=\frac{1}{n}\sum_{i=1}^{n}y_{i},\;\;\;\;\;\;\hat{\bar{y}}=\frac{1}{n}\sum_{i=1}^{n}{\hat{y}}_{i},\;S_{y\hat{y}}=\sum_{i=1}^{n}(y_{i}-\bar{y})({\hat{y}}_{i}-\hat{\bar{y}})\;\;\;S_{yy}=\sum_{i=1}^{n}(y_{i}{-\bar{y})}^{2}\;\;\;\;S_{\hat{y}\hat{y}}=\sum_{i=1}^{n}({\hat{y}}_{i}-{\hat{\bar{y}})}^{2}$$

The applicability domain was evaluated using the leverage approach and visualized by a Williams plot.

The critical leverage value (h*) was calculated as:

$$h^{*}=\frac{3(p+1)}{n}\;\;$$

where *p* is the number of descriptors in the model and *n* is the number of compounds in the training set. Compounds with leverage values below h* and standardized residuals within ±3 were considered within the applicability domain.

## 4. Conclusions

In this study, biomimetic chromatography and machine-learning-based QSRR modeling were integrated to systematically investigate HSA binding in a diverse set of FQs derivatives. The experimentally anchored modeling approach enabled the development of a robust and externally predictive ν-SVR model based on a parsimonious 12-descriptor subset. The model demonstrated strong calibration, cross-validation, and external validation performance, fulfilled OECD validation criteria, and exhibited a well-defined applicability domain. Mechanistic interpretation using SHAP analysis revealed that HSA affinity is primarily governed by a balanced combination of hydrophobic surface area and distributed electronic properties, while excessive localized polarity and quaternization tend to reduce binding. Importantly, the model consistently captured subclass-specific structural effects, reinforcing the chemical relevance and interpretability of the identified structure–binding relationships.

Collectively, the integration of validated predictive performance, mechanistic transparency, and open-access implementation establishes a coherent and transferable framework for rational prediction and optimization of HSA binding within the fluoroquinolone class.

## Figures and Tables

**Table 1:** Predictive performance metrics of the optimized 12-descriptor SVR and ν-SVR models for HSA binding, including calibration, cross-validation, and external validation results.

Metric	SVR	ν-SVR
R^2^	0.901	0.916
MAE	0.161	0.135
RMSE	0.206	0.190
Q^2^_CV_	0.818	0.811
MAE_CV_	0.211	0.204
RMSE_CV_	0.280	0.287
Q^2^_test_	0.800	0.823
MAE_test_	0.194	0.174
RMSE_test_	0.252	0.238
Q^2^_F1_	0.850	0.867
Q^2^_F2_	0.800	0.823
Q^2^_F3_	0.851	0.868
CCC	0.882	0.899

## Data Availability

The original contributions presented in this study are included in the article and Supplementary Materials. Further inquiries can be directed to the corresponding author.

## Supplementary Materials

The following supporting information can be downloaded at: https://www.mdpi.com/article/10.3390/ijms27083700/s1.
